# Improving the predictive potential of diffusion MRI in schizophrenia using normative models—Towards subject‐level classification

**DOI:** 10.1002/hbm.25574

**Published:** 2021-07-29

**Authors:** Doron Elad, Suheyla Cetin‐Karayumak, Fan Zhang, Kang Ik K. Cho, Amanda E. Lyall, Johanna Seitz‐Holland, Rami Ben‐Ari, Godfrey D. Pearlson, Carol A. Tamminga, John A. Sweeney, Brett A. Clementz, David J. Schretlen, Petra Verena Viher, Katharina Stegmayer, Sebastian Walther, Jungsun Lee, Tim J. Crow, Anthony James, Aristotle N. Voineskos, Robert W. Buchanan, Philip R. Szeszko, Anil K. Malhotra, Matcheri S. Keshavan, Martha E. Shenton, Yogesh Rathi, Sylvain Bouix, Nir Sochen, Marek R. Kubicki, Ofer Pasternak

**Affiliations:** ^1^ Department of Mathematics Tel‐Aviv University Tel‐Aviv Israel; ^2^ Department of Psychiatry Brigham and Women's Hospital, Harvard Medical School Boston Massachusetts USA; ^3^ Department of Radiology Brigham and Women's Hospital, Harvard Medical School Boston Massachusetts USA; ^4^ Departments of Psychiatry and Neuroscience Massachusetts General Hospital and Harvard Medical School Boston Massachusetts USA; ^5^ Department of Psychiatry University Hospital, Ludwig Maximilian University of Munich Munich Germany; ^6^ IBM Research AI Haifa Israel; ^7^ Department of Psychiatry Yale University New Haven Connecticut USA; ^8^ Department of Psychiatry UT Southwestern Medical Center Dallas Texas USA; ^9^ Department of Psychiatry and Behavioral Neuroscience University of Cincinnati Cincinnati Ohio USA; ^10^ Departments of Psychology and Neuroscience Bio‐Imaging Research Center, University of Georgia Athens Georgia USA; ^11^ Department of Psychiatry and Behavioral Sciences, Morgan Department of Radiology and Radiological Science Johns Hopkins Medical Institutions Baltimore Maryland USA; ^12^ Translational Research Center University Hospital of Psychiatry, University of Bern Bern Switzerland; ^13^ Department of Psychiatry University of Ulsan College of Medicine, Asan Medical Center Seoul South Korea; ^14^ Department of Psychiatry, SANE POWIC Warneford Hospital, University of Oxford Oxford UK; ^15^ Centre for Addiction and Mental Health, Department of Psychiatry University of Toronto Toronto Canada; ^16^ Maryland Psychiatric Research Center, Department of Psychiatry University of Maryland School of Medicine Baltimore Maryland USA; ^17^ Department of Psychiatry Icahn School of Medicine at Mount Sinai New York New York USA; ^18^ Mental Illness Research, Education and Clinical Center James J. Peters VA Medical Center New York New York USA; ^19^ The Feinstein Institute for Medical Research and Zucker Hillside Hospital Manhasset New York USA; ^20^ Department of Psychiatry, Beth Israel Deaconess Medical Centre Harvard Medical School Boston Massachusetts USA

**Keywords:** diffusion magnetic resonance imaging, machine learning, precision medicine, schizophrenia, white matter

## Abstract

Diffusion MRI studies consistently report group differences in white matter between individuals diagnosed with schizophrenia and healthy controls. Nevertheless, the abnormalities found at the group‐level are often not observed at the individual level. Among the different approaches aiming to study white matter abnormalities at the subject level, normative modeling analysis takes a step towards subject‐level predictions by identifying affected brain locations in individual subjects based on extreme deviations from a normative range. Here, we leveraged a large harmonized diffusion MRI dataset from 512 healthy controls and 601 individuals diagnosed with schizophrenia, to study whether normative modeling can improve subject‐level predictions from a binary classifier. To this aim, individual deviations from a normative model of standard (fractional anisotropy) and advanced (free‐water) dMRI measures, were calculated by means of age and sex‐adjusted *z*‐scores relative to control data, in 18 white matter regions. Even though larger effect sizes are found when testing for group differences in *z*‐scores than are found with raw values (*p* < .001), predictions based on summary *z*‐score measures achieved low predictive power (AUC < 0.63). Instead, we find that combining information from the different white matter tracts, while using multiple imaging measures simultaneously, improves prediction performance (the best predictor achieved AUC = 0.726). Our findings suggest that extreme deviations from a normative model are not optimal features for prediction. However, including the complete distribution of deviations across multiple imaging measures improves prediction, and could aid in subject‐level classification.

## Graphical abstract

We use a large (*n* = 1113) harmonized diffusion MRI dataset of individuals diagnosed with schizophrenia and healthy controls, in order to study the predictive performance of a normative modeling approach. This approach compares each individual with distribution derived from healthy controls to derive measures of deviation from the normal distribution. Our results show that combining deviation information from different white matter tracts, while using multiple imaging measures simultaneously, improves prediction performance, and could therefore aid in subject‐level classification.

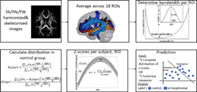

## 1 INTRODUCTION

Aligned with postmortem findings of anomalies in white matter (Coyle, Balu, Puhl, & Konopaske, 2016; Friston, 1998), diffusion MRI (dMRI) studies consistently demonstrate a disturbed white matter structural organization in schizophrenia (Cetin‐Karayumak et al., 2020; Ellison‐Wright & Bullmore, 2009; Kelly et al., 2018; Kubicki et al., 2007; Skudlarski et al., 2013). For example, the largest, multisite case–control analysis of dMRI measures in schizophrenia to date, Kelly et al. (2018), observed significantly lower fractional anisotropy (FA) (Basser, Mattiello, & LeBihan, 1994), in the schizophrenia group, in 20 of 25 white matter regions examined.

The vast majority of dMRI studies in schizophrenia apply case–control comparisons between individuals diagnosed with schizophrenia and healthy controls to identify significant group‐level differences in specified white matter locations. However, group differences that are found in a case–control comparison do not imply abnormalities in a given individual subject (see e.g. Arbabshirani, Plis, Sui, & Calhoun, 2017). For example, the hallmark finding of widespread FA reductions in the schizophrenia group (Kelly et al., 2018), does not necessarily imply that widespread FA reductions are present in every individual diagnosed with schizophrenia, although an implicated location may be present in a subset of individuals. This highlights the need for alternative analysis paradigms that can better account for individual variation in pathological loci.

There are two leading analysis methods that provide subject specific inferences: The first is prediction modeling, which aims to classify each subject into one of several groups, thereby making it more suitable for clinical diagnosis. The second is normative modeling, which aims to characterize individual variations in reference to a normative range. Unlike the case–control approach that searches for group differences in the mean value of some feature in a specific brain location (e.g., mean FA in one specific white matter tract), prediction approaches search for features that maximize the separation between the groups. Separation is usually measured by the *area under the receiver operator curve* (AUC) of a particular prediction classifier. Previous studies (see e.g. Ardekani et al., 2011; Lee et al., 2018; Mikolas et al., 2018; Rathi et al., 2010 and the references therein) have already demonstrated that dMRI measures can serve as discriminative features in the discrimination of individuals diagnosed with schizophrenia from healthy controls, but suffered from relatively small sample sizes, which questions the generalizability of their results.

Normative modeling is an alternative paradigm, based on the notion that different individuals could be affected by different patterns of abnormality. In normative modeling, the range of variation within the control group is modeled first, and then individual deviations from this range are calculated, providing information about potential abnormalities in each particular individual. This is different from the case–control approach, which assumes a consistent pattern of abnormality across individuals that belong to the same group. Deviations are typically quantified using a *z*‐score, relative to the control group, and abnormalities are identified as those values that are outliers relative to the distribution of the control group, that is, having *z*‐scores with an absolute value larger than a threshold (Bouix et al., 2013; Marquand et al., 2019; Marquand, Rezek, Buitelaar, & Beckmann, 2016). The ability of the normative modeling approach to shed light on individualized abnormality profiles was leveraged by studies applying normative modeling on various neuroimaging datasets, often to investigate heterogeneity of abnormalities across subjects. Studies applying normative modeling on diffusion MRI are available, for example, in traumatic brain injuries (Bouix et al., 2013; Pasternak et al., 2014; Taylor, da Silva, Blamire, Wang, & Forsyth, 2020), autism and brain development (Chamberland et al., 2020; Dean III et al., 2017; Dimitrova et al., 2020). A few studies have also applied normative modeling on data from subjects diagnosed with schizophrenia, using diffusion MRI (Lv et al., 2020; White, Schmidt, & Karatekin, 2009) and T1‐weighted MRI (Alexander‐Bloch et al., 2014; Wolfers et al., 2018, 2021). References to more studies applying normative modeling on different datasets can be found in Marquand et al. (2019).

The few published normative modeling studies applied on subjects diagnosed with schizophrenia, using diffusion MRI (Lv et al., 2020; White et al., 2009), or T1‐weighted MRI (Wolfers et al., 2018, 2021), found high interindividual differences in the locations of the implicated brain abnormalities. In a recent study, applying normative modeling on diffusion MRI data (Lv et al., 2020), it was further shown that the majority of individuals with schizophrenia had at least one abnormal location implicated, when considering FA as the modality of choice. At the same time, however, a large number of healthy controls also showed at least one abnormal location.

While normative modeling aims to provide useful insights at the subject‐level, previous studies did not utilize the framework to go beyond group‐level differences between the schizophrenia and control groups. In this article, we use a large sample of harmonized dMRI data (Cetin‐Karayumak et al., 2020), comprised of 512 healthy controls and 601 individuals diagnosed with schizophrenia, to evaluate the predictive power of features derived from a normative modeling approach and compare it with the predictive power of raw dMRI values serving as features. Here, our motivation is to improve the characterization of the schizophrenia group as a whole by assuming that common abnormalities (e.g., decreased FA/FAt, increased FW) may occur in spatially distinct regions across subjects. By using the features obtained from the normative model in a classification scheme, we test whether these profiles provide an improved characterization of the group, compared to the raw values.

We emphasize that as the diagnosis of schizophrenia relies upon identifying several different combinations of clinical symptoms and behavioral signs through an interview with a medical specialist, we do not expect that combining the normative modeling approach with classification would yield a performance that is comparable to clinical diagnosis. Rather, our aim is to provide new information about white matter abnormalities in schizophrenia using the combination of the two approaches, which may be proven useful in the future design of classification schemes for the diagnosis of schizophrenia.

Previous studies utilizing this dataset have already demonstrated significant group‐differences in FA across the life span between healthy controls and individuals diagnosed with schizophrenia, as well as age effects (Cetin‐Karayumak et al., 2020), and sex effects in healthy controls (Seitz et al., 2020). Here, we take a step towards subject‐level inferences by investigating the application of the normative modeling approach on this dataset. We first generate a normative model by estimating age‐ and sex‐adjusted *z*‐scores from standard (FA) and advanced (Free‐water) dMRI measures in 18 white matter regions of interest (ROIs). Then, for every subject, the predictive performance of the following features is calculated and compared with the predictive performance of the raw dMRI values: (1) *z*‐scores obtained by applying the normative modeling approach on FA values; (2) summary measures for the *z*‐score distributions (Pasternak et al., 2014); (3) *z*‐scores and summary measures obtained by applying the normative modeling approach on free‐water imaging derived measures (Pasternak, Sochen, Gur, Intrator, & Assaf, 2009) rather than on FA.

## 2 MATERIALS AND METHODS

### 2.1 Participants, imaging acquisition, image preprocessing and harmonization procedures

The dataset used in this study coincides with the dataset utilized in the published work by (Cetin‐Karayumak et al., 2020), which includes 601 individuals diagnosed with schizophrenia‐spectrum disorder across multiple illness stages (mean [SD] age, 31.46 [12.31] years; 380 [63.23%] male), and 512 healthy controls (mean [SD] age, 30.15 [14.26] years; 279 [54.49%] male). dMRI data were collated from 13 different sites across a number of separate studies. The single shell dMRI data followed a standardized preprocessing protocol and were harmonized across sites to remove site‐related differences using retrospective harmonization (Karayumak et al., 2019; Ning et al., 2020). In particular, Cetin‐Karayumak et al. (2020) evaluated the performance of the harmonization procedure by using unpaired *t* tests to assess between‐site differences and showed that statistical differences between matched controls across sites were removed after harmonization (see Figure S2 in Cetin‐Karayumak et al., 2020). We note that following the harmonization, site differences between subjects diagnosed with schizophrenia are likely to occur, because of different distributions across sites of parameters such as age, sex, and type of clinical populations. These differences are important to be preserved, as they reflect true variability related to the disorder, while scanner related differences are removed. A complete account of demographics, inclusion and exclusion criteria, acquisition protocols across the 13 sites, preprocessing and harmonization procedures can be found in Cetin‐Karayumak et al. (2020). Following harmonization, all data had isotropic resolution of 1.5 mm × 1.5 mm × 1.5 mm, with a *b*‐value of 1,000 s/mm^2^.

### 2.2 White matter processing

The harmonized data were fitted using FSL's DTIFIT (Behrens et al., 2003) to the DTI model, from which FA was derived. The data were also fitted to the two‐compartments Free‐water imaging model (including a free‐water compartment and a tissue compartment) using a regularized nonlinear fit (Pasternak et al., 2009). In this process, the fractional volume of the free‐water compartment (FW) as well as the FA of the tissue compartment (FAt) were estimated, as previous work suggests that these may increase sensitivity to underlying pathologies (Lyall et al., 2018; Pasternak et al., 2012; Pasternak, Westin, Dahlben, Bouix, & Kubicki, 2015).

To define white matter regions of interest (ROIs) we used the IIT Human Brain probabilistic white matter fiber tract ROIs atlas v. 4.1 (Varentsova, Zhang, & Arfanakis, 2014) with a threshold of 0.25, resulting in a total of 17 white matter fiber tract ROIs. The FA image of each subject was registered to the FA IIT template using ANTs registration (Avants et al., 2011), and this transformation was applied to the other diffusion measures (FAt, FW). For each tract, mean FA, FAt and FW were computed across all voxels traversing the fiber bundle. Since the IIT atlas v.4.1 that we used does not cover all of the white‐matter, we complemented the analysis by computing the white matter skeleton averaged FA, FAt and FW across voxels comprising the IIT white matter skeleton template (IIT_WM_atlas_skeletonized.nii.gz) (Varentsova et al., 2014).

### 2.3 Construction of a normative model

The normative model represents the distribution of the normative range within each ROI in the healthy controls using the sample mean and standard deviation. To control for confounding factors resulting from age and sex differences, we represented the normative range in each ROI by an age specific weighted mean, $\hat{m_{h}}$, and standard deviation, ${\hat{\sigma }}_{h}^{2}$, for each sex separately. To do so, we used the Nadaraya‐Watson (NW) estimator (Nadaraya, 1964; Watson, 1964) with a Gaussian kernel,

(1a) $$\hat{m_{h}}\left(x\right)=\frac{\sum_{i=1}^{n}y_{i}K\left(\frac{x-x_{i}}{h}\right)}{\sum_{i=1}^{n}K\left(\frac{x-x_{i}}{h}\right)},$$

(1b) $${\hat{\sigma }}_{h}^{2}\left(x\right)=\frac{\sum_{i=1}^{n}{\left(y_{i}-\hat{m_{h}}\left(x\right)\right)}^{2}K\left(\frac{x-x_{i}}{h}\right)}{\sum_{i=1}^{n}K\left(\frac{x-x_{i}}{h}\right)},$$

where x is the patient age and n is the size of the sex‐matched control group. For the *i*th individual in the sex‐matched control group, $y_{i}$ is the dMRI value (e.g., the mean FA value over the ROI), and $x_{i}$ is the age. $K\left(u\right)=\frac{1}{\sqrt{2\pi }}e^{-\frac{1}{2}u^{2}}$ is a Gaussian kernel, and h>0 is a bandwidth parameter. To set h for every ROI, and every dMRI modality (FA, FAt, or FW), we minimized the cross‐validation function,

(2) $$\mathrm{CV}\left(h\right)=\frac{1}{n}\sum_{j=1}^{n}{\left\{y_{j}-\hat{m_{h,-j}}\left(x_{j}\right)\right\}}^{2},$$

where $\hat{m_{h,-j}}$ is the leave‐one‐out‐estimator,

$$\hat{m_{h,-j}}\left(x\right)=\frac{\sum_{i\neq j}y_{i}K\left(\frac{x-x_{i}}{h}\right)}{\sum_{i\neq j}K\left(\frac{x-x_{i}}{h}\right)}.$$

The procedure therefore guarantees that we select the bandwidth for which the weighted mean $\hat{m_{h}}$ best reflects the normative range. The chosen bandwidths are reported in Table S1.

### 2.4 Calculation of deviation from the normative model

The deviation of every individual diagnosed with schizophrenia from the normative atlas, in each ROI, was captured by a *z*‐score, calculated using the NW estimators $\hat{m_{h}},{\hat{\sigma_{h}}}^{2}$ (see Equations (1a) and (1b)),

$$z\left(x\right)=\frac{y-\hat{m_{h}}\left(x\right)}{{\hat{\sigma }}_{h}\left(x\right)},$$

where x is the subject's age and y is the subject's dMRI value (e.g., the mean FA value over the ROI). The *z*‐scores were truncated to the range $\left[-10,10\right]$. The same procedure was also used to evaluate deviation of each healthy control subject, but with a leave‐one‐out approach, that is, we compared a given healthy control subject with a normative model composed of all healthy control subjects, excluding the one being evaluated. As a result, for each subject, and for each dMRI value (FA, FAt, or FW), we obtained a vector with 18 *z*‐scores (for 17 tracts + white matter skeleton) representing deviation from the normative model.

**FIGURE 1:**
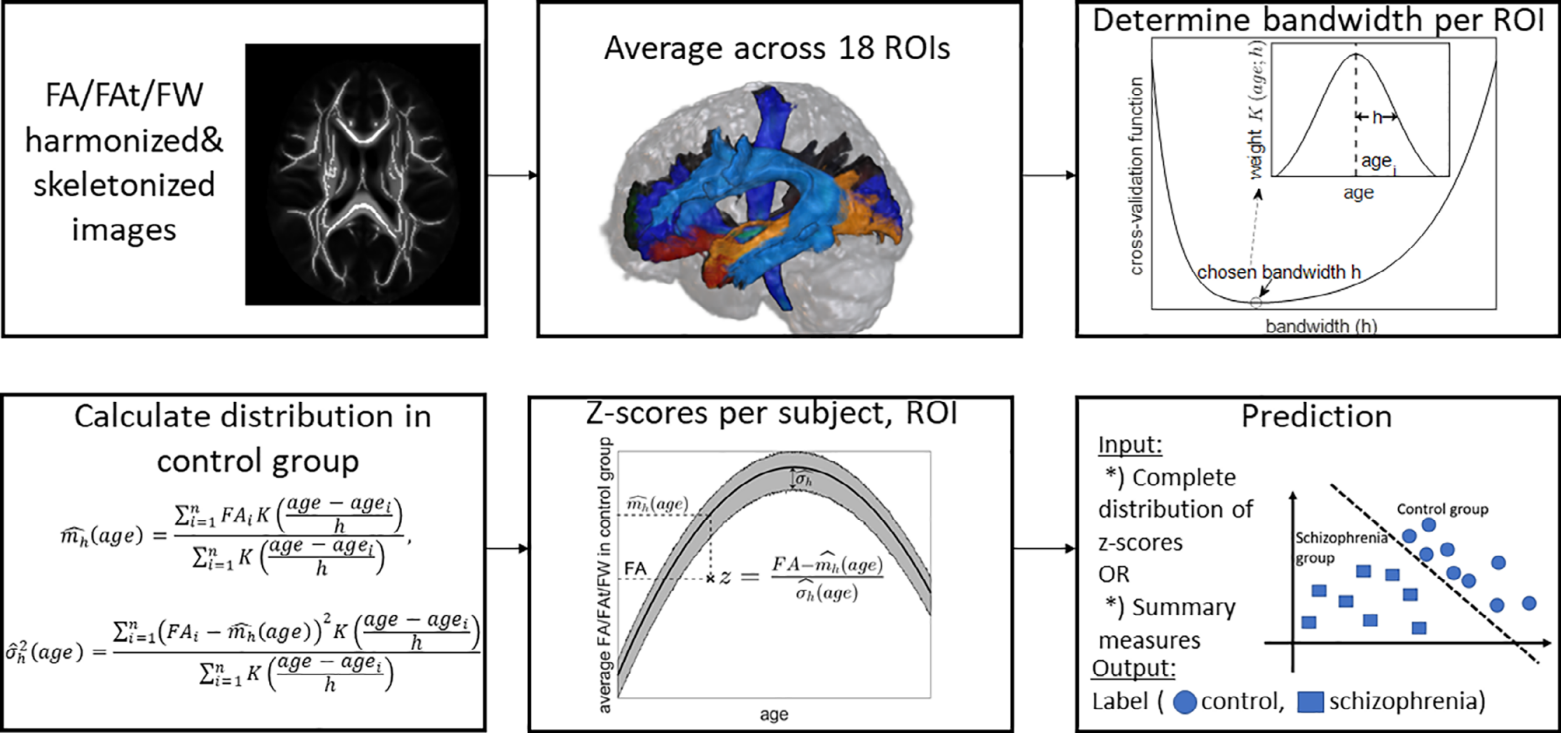
A flowchart summarizing the analysis scheme. The details are provided in the text

Our approach is summarized in Figure 1, as well as in Algorithm 1.

Algorithm 1 **Calculation of FA *z*‐scores for every subject**

**// Calculate bandwidth and weight function for each ROI**

**for each** ROI R **do**:

 h[R] ← minimizer of cross‐validation function (Equation (2)) calculated using FA values of control group in ROI R

**// Calculate *z* scores for subjects diagnosed with schizophrenia**

**for each** “subject diagnosed with schizophrenia” s **do**:

 age_s ← age of subject s

 sex_s ← sex of subject s

 
**for each** ROI R **do**:

    FA[1,…,*n*] ← FA values in ROI R of all controls of sex_s

    age[1,…,*n*] ← ages of all controls of sex sex_s

    
**// Calculate mean and standard deviation in controls centered at age_s**

    FA_s ← FA value in ROI R of subject s

    Mean ← weighted_mean(FA,FA_s,age,age_s,h[R]) using Equation (1a)

    Std ← weighted_std(FA,FA_s,age,age_s,h[R]) using Equation (1b)

    
**// Calculate *z*‐score for subject s**

    Z_scz[s] ← $\frac{\mathrm{FA}\_s-\text{Mean}}{\mathrm{Std}}$

**// Calculate *z* scores for control subjects**

**for each** control subject c **do**:

 age_c ← age of subject c

 sex_c ← sex of subject c

 
**for each** ROI R **do**:

    FA[1,…,*m*] ← FA values in ROI R of all controls of sex sex_c excluding control c

    age[1,…,*m*] ← ages of controls of sex sex_c excluding control c

    FA_c ← FA value in ROI R of control c

    Mean ← weighted_mean(FA,FA_s,age,age_s,h[R]) using Equation (1a)

    Std ← weighted_std(FA,FA_s,age,age_s,h[R]) using Equation (1b)

    Z_HC[c] ← $\frac{\mathrm{FA}\_c-\text{Mean}}{\mathrm{Std}}$

return (Z_scz,Z_HC)

### 2.5 Group‐level differences in ROI‐wise values

Group comparisons of raw dMRI values (i.e., the FA, FAt and FW values before the construction of the normative model) and *z*‐score values (for FA, FAt and FW) of all subjects in each ROI were performed using 1‐tailed Welch's *t* tests (Welch, 1951) searching for lower FA and FAt values and higher FW values in the schizophrenia group. Welch's *t* test inherently accounts for possible unequal variance or sample size in the two compared groups, and is equivalent to the Student's *t* test whenever sample size and variance in the two compared groups are equal (Delacre, Lakens, & Leys, 2017). We also report Cohen's *d* effect size (Cohen, 2013) for every hypothesis test. To allow comparisons with subsequent tests, we also used 1‐tailed two‐sample Wilcoxon ranks sum tests.

### 2.6 *z*‐score derived summary measures

To define abnormal *z*‐scores we used the threshold of |*z*| > 2.999, corresponding to *p* < .05 Bonferroni corrected for 18 tests (for 18 ROIs). ROIs with *z*‐scores above 2.999 were defined as supra‐normal, ROIs with *z*‐scores below −2.999 were defined as infra‐normal. To identify if a particular ROI is implicated, for each ROI we counted how many times it is found abnormal across the entire schizophrenia group. To account for a possible heterogeneity in the abnormality location in different subjects, we derived for each subject *z*‐score summary measures that are indifferent to the spatial location of the abnormality. The summary measures included: fraction of abnormal ROIs (also called “load” [Bouix et al., 2013]), *z*‐score with the largest absolute value (also called “severity” [Bouix et al., 2013]), average *z*‐score, standard‐deviation of *z*‐scores and fraction of ROIs having *z*‐scores in the significant range (see below for a definition of the significant range). Since the distribution of the “load” measure is skewed and strongly deviate from the normal distribution in both groups, we used 1‐tailed two‐sample Wilcoxon rank sum tests to perform group comparisons of all summary measures. We also report Cliff's delta effect size (Cliff, 1993) for every hypothesis test. Cliff's delta effect size estimates the difference between two probability scores: (1) the probability that a value selected from one of the groups is greater than a value selected from the other group, and (2) the probability of the reverse case. This test is nonparametric and based on the ordinal structure of the data, which is appropriate for data distributions that deviate from normal.

### 2.7 *z*‐distribution

To better focus on the range of *z*‐scores that best discriminates individuals diagnosed with schizophrenia from healthy controls, the distribution of *z*‐scores was estimated for each subject by collecting the *z*‐scores in all ROIs and computing the probability density function (PDF), regularized by a normal distribution kernel, in 50 equally spaced bins that cover the range (−10,10). We then compared the PDFs between the healthy controls and the schizophrenia groups by comparing the density in each bin, using a 1‐tailed Welch's *t* test searching for higher values in the schizophrenia group. This comparison provided a range of *z*‐scores (referred to as the *significant range*) which appear significantly more frequently in the schizophrenia group than in the healthy controls group.

### 2.8 Prediction models

We examined the diagnostic potential of the normative modeling approach by using the *z*‐score maps, as well as the *z*‐score derived measures, as inputs to a binary classifier, with the aim of classifying individual subjects as either healthy controls or as diagnosed with schizophrenia. In comparison, we also built binary classifiers with raw dMRI values as the input. We chose logistic regression with ridge regularization (McIlhagga, 2016) as the binary classifier of choice, thus enforcing sparse and stable classification solutions. Explicitly, we examined the following measures as inputs to the classifier: (1) FA/FAt/FW raw values in each ROI separately, (2) FA/FAt/FW *z*‐score values in each ROI separately, (3) FA/FAt/FW *z*‐scores in all ROIs simultaneously (concatenated into one vector of length 18 for each dMRI measure), (4) FAt and FW *z*‐scores in all ROIs simultaneously (concatenated into one vector of length 36 for each subject), and (5) combination of summary measures and the aforementioned inputs.

Prediction performance of the estimated models was validated using a 10‐fold cross‐validation procedure. The data were partitioned into 10 subsets—seven subsets comprised of 51 subjects from the control group and 60 subjects from the schizophrenia group, two subsets comprised of 52 subjects from the control group and 60 subjects from the schizophrenia group, and one subset comprised of 51 subjects from the control group and 61 subjects from the schizophrenia group. In each cross‐validation round, one of the 10 subsets served as the test set, while the other 9 subsets served as the training set for the binary classifier. The average of the area under the receiver operator curve (AUC), across the 10 test sets, was the evaluation metric. We note that in each cross‐validation, the normative range, as well as the choice of a bandwidth, were estimated using only the healthy control subjects that belonged to the corresponding training set. This guaranteed that the classification performance on the test sets was not biased by the estimated normative model.

In order to examine whether sex differences exist, we have repeated the same process (including the choice of a bandwidth) for males and females separately.

## 3 RESULTS

### 3.1 Group‐level differences in ROI‐wise values

**FIGURE 2:**
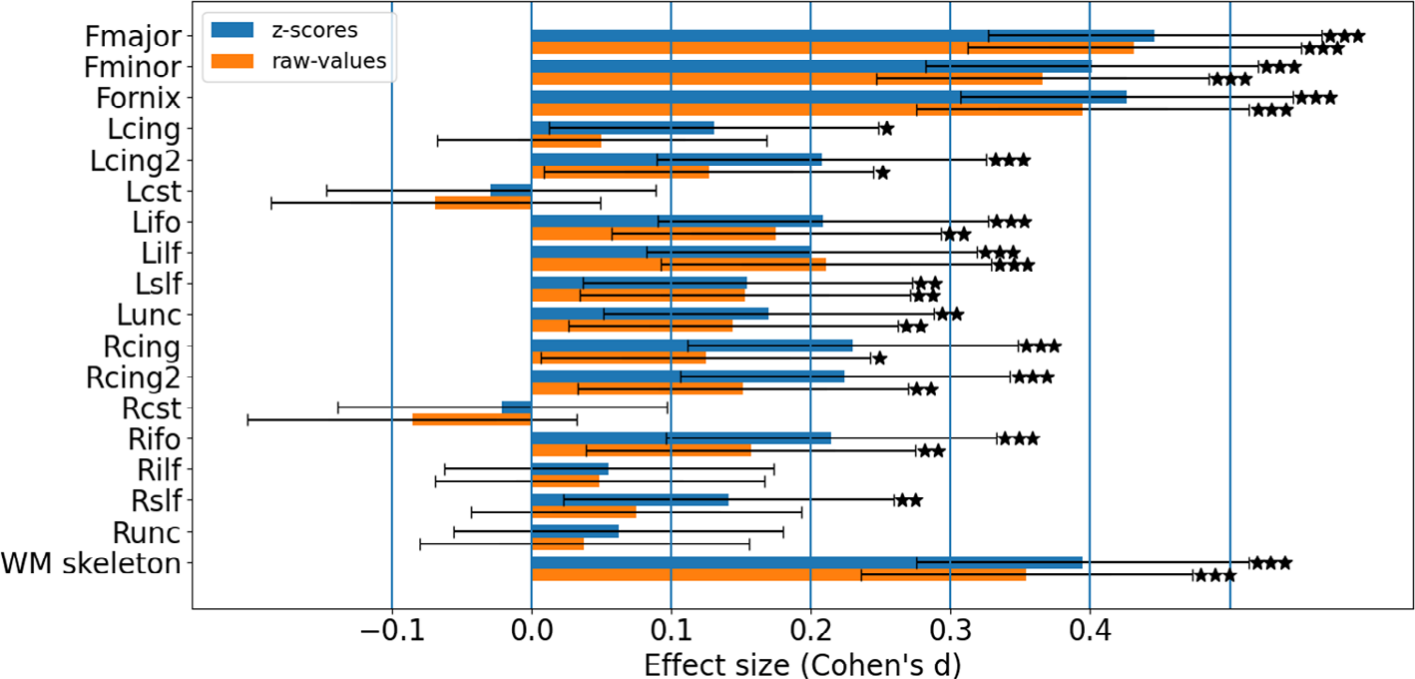
Group differences in raw and *z*‐score FA values. The plots display effect sizes obtained when testing for lower raw FA values in the schizophrenia group (orange bars) or lower FA *z*‐scores (blue bars). Most ROIs showed significant group differences in both raw and *z*‐score values, although the effect sizes for the *z*‐scores were higher than for the raw values. The full ROI names are detailed in supplementary material. Error bars represent 95% confidence interval for Cohen's‐*d* effect size. Group difference *p*‐values: ★ .01 < *p* < .05, ★★ .001 < *p* < .01, ★★★ *p* < .001

Group comparisons of the raw FA values of the 18 ROIs between the healthy controls group and the schizophrenia group identified significantly lower FA values in the schizophrenia group in 12/18 ROIs (Figure 2 and Table S2), which is consistent with previous case–control studies in schizophrenia (Kelly et al., 2018; Lv et al., 2020; Wolfers et al., 2018 and the references therein) and studies using the same dataset as ours (Cetin‐Karayumak et al., 2020). Group comparisons between the *z*‐scores of the FA values identified significant differences in 14/18 ROIs. Of note, the effect sizes for group‐differences were higher (*p* < .001 using a one‐sided paired *t* test; Cohen's *d* = 0.294) when testing for differences in *z*‐scores, compared with testing for differences in the raw FA values **(**Figure 2 and Figure S1 **)**.

### 3.2 Subject specific *z*‐score derived summary measures

The ROI with the highest occurrence of infra‐normal *z*‐values (*z* < **−**2.9913) was the Forceps major (Fmajor), found in only 19/601 (3.16%) individuals diagnosed with schizophrenia (Table S3). In addition, 62/601 (10.3%) of the individuals diagnosed with schizophrenia had at least one infra‐normal ROI, compared to 37/512 (7.2%) of the healthy controls.

**FIGURE 3:**
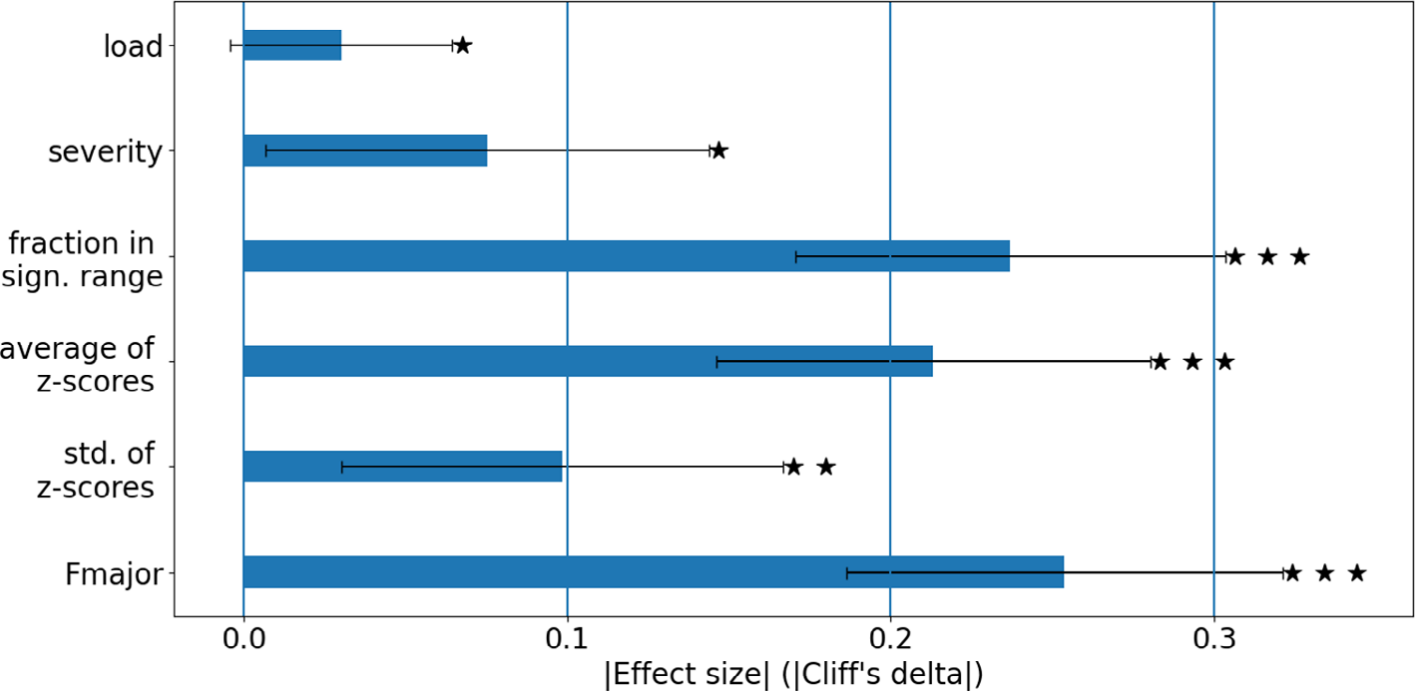
Summary measures. The plots present effect sizes (in absolute values) for group differences in each of the summary measures. For comparison, the effect size obtained when using only the value for the Forceps major is included. sign = significant, std = standard deviation, Fmajor = Forceps Major. Error bars represent 95% confidence interval for Cliff's‐delta effect size (Feng & Cliff, 2009). Group difference *p*‐values: ★ .01 < *p* < .05, ★★ .001 < *p* < .01, ★★★ *p* < .001

All *z*‐score derived distribution summary measures showed a significant group‐difference with varying effect sizes (Figure 3). These measures included load (*p* = .039; Cliff's delta = −0.03), severity (*p* = .015; Cliff's delta = −0.075), average *z*‐score across all ROIs (*p* < .001; Cliff's delta = 0.213), and standard deviation of *z*‐score values (*p* = .002; Cliff's delta = −0.098).

Testing what range of FA *z*‐scores best discriminates the schizophrenia group from the control group identified the range of −3.36 < *z* < −0.6, corresponding to lower FA values in the schizophrenia group. This range only partially overlaps with the infra‐normal range of *z* < −2.99. In addition, the majority of the values within this range are well within what is considered the “normal” range (|*z*| < 2.99). Identifying the fraction of fiber tracts with values in the significant range had higher effect size than using any of the other summary measures (fraction in significant range, *p* << .001; Cliff's delta = −0.2369). However, we note that effect sizes for the summary measures were smaller than those for the group differences of the average *z*‐score in individual tracts (e.g., Fmajor *p* < .001; Cliff's delta =0.25), see Figure 3.

### 3.3 Prediction models

**FIGURE 4:**
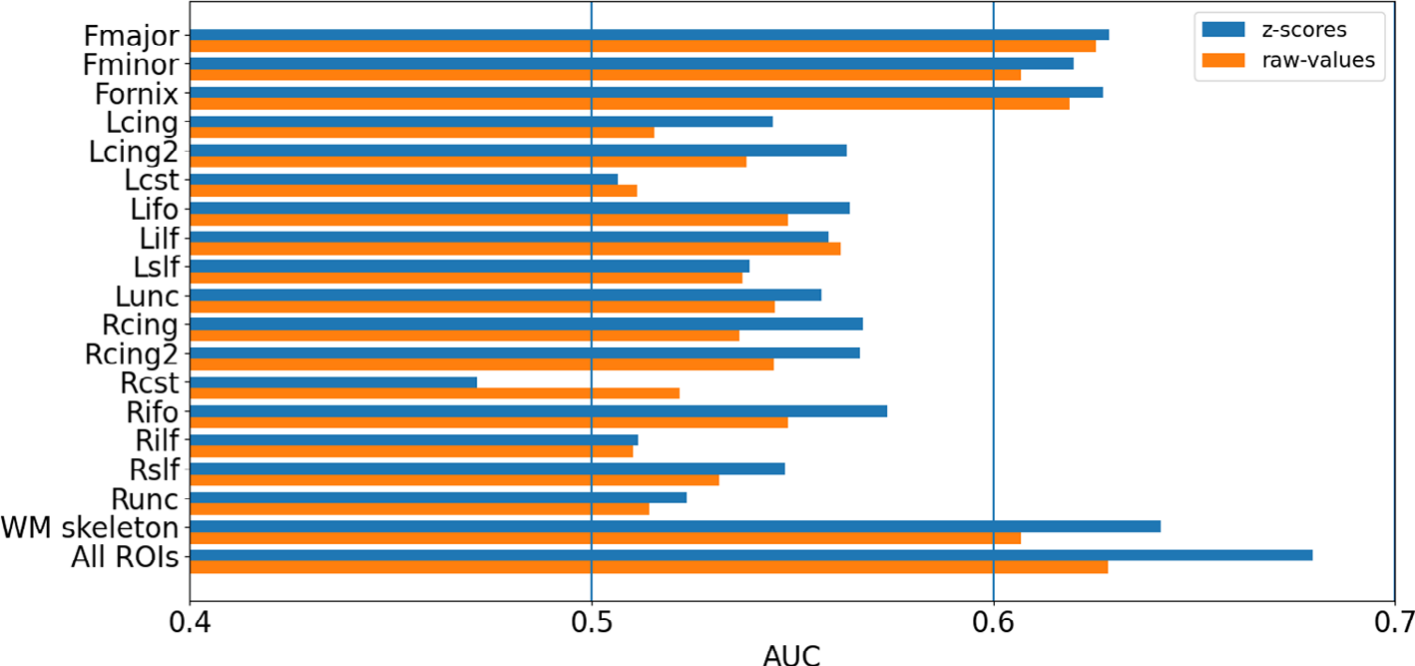
Prediction power for individual ROIs and ROIs combined. Prediction power is reported as area under the receiver–operator curve (AUC), averaged over the cross‐validations in each ROI. AUC is reported for *z*‐scores (blue bars) and for raw values (orange bars). The full ROI names are detailed in supplementary material

The use of the raw FA value or the *z*‐score value for each ROI individually as input for a prediction classifier, resulted in relatively low predictive performance (Figure 4). In the majority of tracts (15/18), the mean AUC (averaged across the cross validations) obtained for the *z*‐score values as input to the classifier was higher than the mean AUC for the raw values as input. Of these, the best predictors were the *z*‐scores of the WM skeleton average (AUC = 0.64), followed by the Forceps Major (Fmajor, AUC = 0.627), Fornix (AUC = 0.627), and the Forceps Minor (Fminor, AUC = 0.621) ROIs. Importantly, using the *z*‐scores of all ROIs simultaneously as input to the binary classifier resulted in a higher predictive power than any individual ROI (Figure 4), yielding an AUC of 0.67. Inclusion of the subject specific summary measures to the *z*‐scores in all the other ROIs did not improve the AUC.

### 3.4 Multiple imaging features

Upon repeating the analyses for the FAt and FW measures derived from free‐water imaging (Table S2), we found that the number of individuals diagnosed with schizophrenia who had infra‐normal FAt or supra‐normal FW values was higher than the number of individuals diagnosed with schizophrenia who had infra‐normal FA. At the same time, the number of healthy controls with abnormal FAt or FW did not increase compared to our FA analyses (Table S3). Specifically, 87/601 (14.47%) of the individuals diagnosed with schizophrenia had at least one ROI with an infra‐normal FAt value, compared to 38/512 (7.42%) of the healthy controls. Similarly, 84/601 (13.97%) of the individuals diagnosed with schizophrenia had at least one ROI with a supra‐normal FW value, compared to 35/512 (6.8%) of the healthy controls. Similar to FA, regions in the *z*‐distributions which exhibited group differences extended within what is considered the “normal” range, exhibiting lower FAt values (−4.3 < *z* < −0.6) and higher FW values (0.97 < *z* < 3.08) in the schizophrenia group compared to the healthy control group.

**FIGURE 5:**
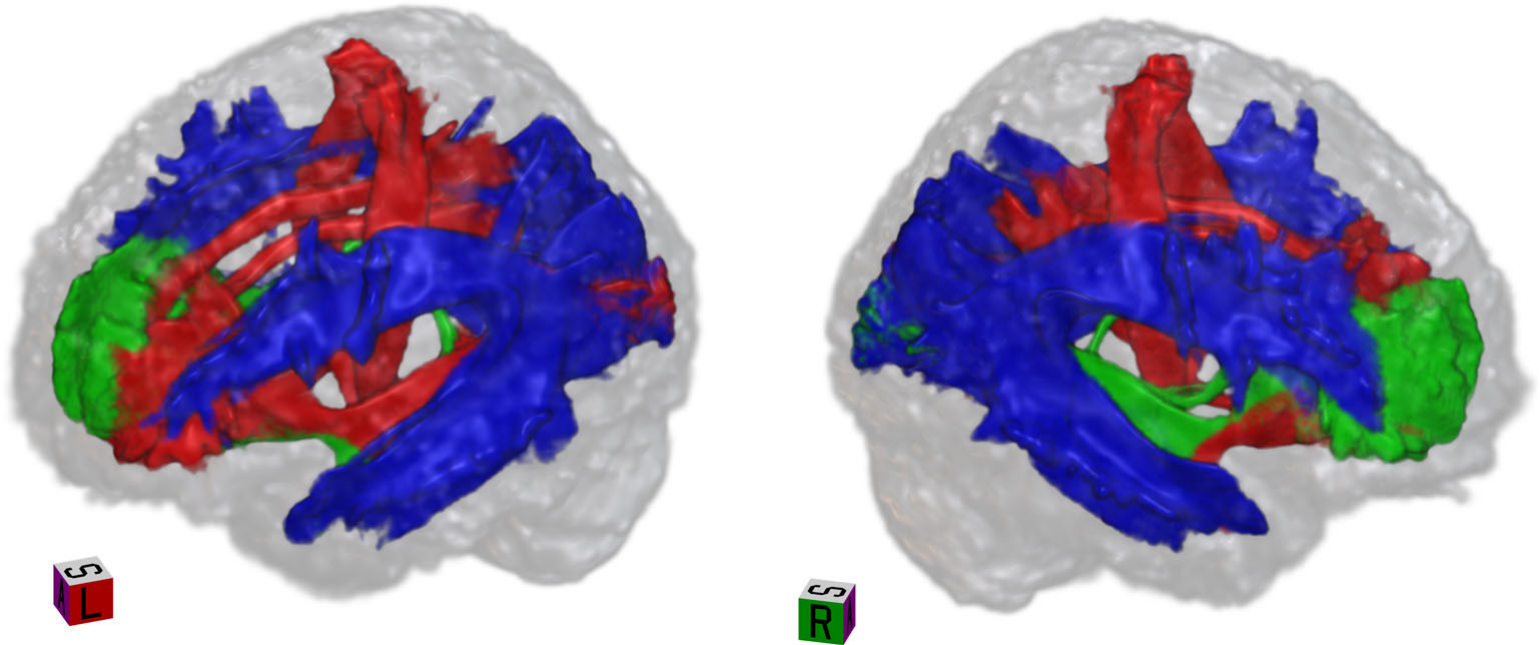
Strongest predictors per dMRI modality. Each ROI is colored according to the dMRI modality (FA in green, FAt in red, and FW in blue) that had the highest AUC for classification

**FIGURE 6:**
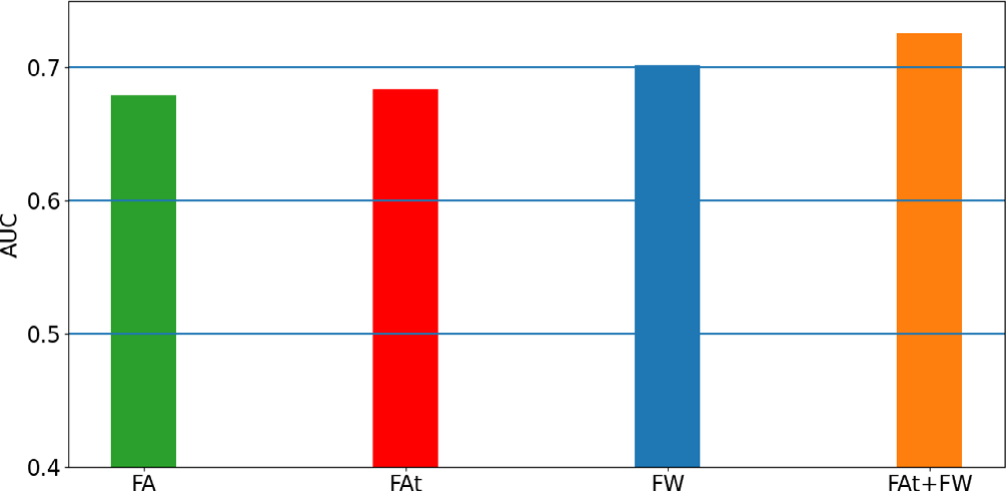
Prediction power for dMRI modalities. Area under the receiver–operator curves (AUC), averaged over the cross‐validations, obtained when inputting the values in all ROIs simultaneously into the classifier, for FA (green bars), FAt (red bars), FW (blue bars) and FAt+FW (orange bars)

Compared with the FA analyses, the individual ROI analyses had higher AUC scores in either FAt or FW: FAt had the highest AUC in 8/18 ROIs, FW in 5/18 ROIs and FA in 5/18 ROIs (Figure 5). Additionally, when inputting the *z*‐scores of all the fiber tracts simultaneously into the binary classifier, both FAt and FW had higher AUC than FA, reaching an AUC of 0.68 and 0.7, respectively (Figure 6 and Figure S2). The highest score (AUC = 0.726) was achieved when inputting together all the *z*‐scores of all ROIs for both the FAt and the FW measures into the classifier (Figure 6 **)**. We note that the largest regression coefficients (averaged across cross‐validations) were assigned to FW across the WM skeleton, FAt across the WM skeleton and FW in Fmajor (see also Figure S3).

When repeating the analysis for males and females separately, we observe that in males, the complete distribution of *z*‐scores in FAt achieved higher score (AUC = 0.6958) than the complete distribution of *z*‐scores in FW (AUC = 0.6641), whereas the opposite was observed in females (AUC = 0.6611 in FAt, AUC = 0.771 in FW), see Figure S4. We also observe that in males, prediction using the *z*‐scores in all ROIs for both FAt and FW as input resulted in better performance (AUC = 0.71) than predictions using the individual dMRI measures (AUC when using FAt = 0.69, AUC when using FW = 0.66), whereas in females, prediction using both FAt and FW resulted in comparable performance (AUC = 0.77) to the performance obtained when only using FW (AUC = 0.77), but higher than the performance obtained when using FAt (AUC = 0.66), see Figure S4. We also note that while the largest regression coefficients in males were assigned to FW across the WM skeleton ROI and FAt across the WM skeleton ROI, the largest regression coefficients in females were assigned to FW in Fmajor, FW in Fminor and FW across the WM skeleton ROI.

## 4 DISCUSSION

In this article, we demonstrate the predictive potential of the normative modeling approach. Our key finding is that the use of the complete distribution of deviations from the normative range of each individual as an input to a binary classifier improves the predictive performance for all tested measures (FA, FAt, FW). Even though we only reached a performance level indicative of an “acceptable discrimination” (c.f., p. 162 in Hosmer Jr, Lemeshow, & Sturdivant, 2013), our findings can serve as an early step in the development of a classification scheme that involves schizophrenia and therefore aid in subject‐level classification.

We also find that extreme deviations from the normative model are not found in a sufficient number of individuals diagnosed with schizophrenia, and, accordingly, summary measures based on extreme deviations are less efficient diagnostic measures. Indeed, the *z*‐distribution analysis identified that the range of *z*‐scores that best discriminates the individuals diagnosed with schizophrenia from controls is bounded and does not include the most extreme range of *z*‐scores. This strongly suggests that extreme *z*‐scores may not be indicative of schizophrenia related pathologies, but rather of other effects such as noise, imaging artifacts, or medication effects (Meng et al., 2019).

Instead of focusing on summary measures of extreme *z*‐scores, we find that the complete distribution of deviations, and their combined effect on a number of imaging measures provides a more solid basis for prediction algorithms, also suggesting that underlying pathologies in schizophrenia are likely subtle and diverse. We emphasize that since our evaluation metric (AUC) is computed on the different test sets, rather than on the training sets, it is not a priori expected that the inclusion of more features will necessarily result in an improved prediction (Guyon & Elisseeff, 2003). In particular, adding features that are irrelevant (e.g., random noise) or redundant (e.g., correlated with one of the already present features) is not expected to improve the predictive performance and may worsen the model generalizability by increased overfit (Guyon & Elisseeff, 2003; Veronese, Castellani, Peruzzo, Bellani, & Brambilla, 2013; Ying, 2019). The finding of an improved predictive performance when using the complete deviation distribution across multiple white matter ROIs therefore highlights the non‐localized nature of white matter abnormalities in schizophrenia.

Similar to our findings, three previous studies that applied normative modeling on schizophrenia datasets (Lv et al., 2020; Wolfers et al., 2018, 2021) also found that considering each ROI separately identifies only a small fraction of subjects as abnormal. These results suggest biological heterogeneity in the location of abnormalities across different subjects. Our results, however, further suggest that location heterogeneity is not the only factor underlying abnormalities across the schizophrenia group, but rather that the interplay between individual deviations across different brain location is also involved. This finding coincides with previous studies that highlight the importance of the relationship between different fiber tracts involved with schizophrenia (Gheiratmand et al., 2017; Klauser et al., 2017). Moreover, compared with the previous normative modeling studies, we find a smaller fraction of subjects with at least one “abnormal” ROI. This can be attributed to differences in the dataset sizes, normative range models, confounders control schemes, and abnormality threshold, affecting the quality of prediction. We note, however, that the previous studies did not investigate the potential use of the individual deviation measures in the context of subject‐level predictions. These studies also did not compare the performance of individual deviation maps with raw values in the context of group‐differences and did not consider inclusion of multiple dMRI measures into their analysis.

It is further instructive to examine our manuscript in light of three criteria suggested in Marquand et al. (2019) for the categorization of different normative modeling approaches. The first criterion is the choice of covariates and response variables. In our approach, age is the only covariate, while the response variable is one of several diffusion MRI measures in each white matter ROI. Even though sex is not treated as an additional covariate, it is explicitly accounted for by estimating sex‐specific normative models. The second criterion is based on the chosen way to separate different sources of variation, and in particular to differentiate between variation across participants from variation due to parameter and model uncertainty. In light of this criterion, our normative model is effectively nonlinear and nonparametric, and controls for the degree of uncertainty by the choice of a bandwidth that minimizes the leave‐one‐out cross‐validation error. This is comparable with previous nonparametric approaches for age‐adjustment. The third criterion suggested in Marquand et al. (2019) is the degree of individual prediction provided by the normative model. This criterion deals with the ability of the normative model to perform single‐subject inferences. In contrast to normative modeling approaches that only provide numerical deviations from the normative model (Cole & Franke, 2017; Marquand et al., 2019). Our model also accounts for the variance within the healthy control group, when providing individual inferences, and therefore allows to estimate the statistical significance of each individual deviation from the normative range. We also compute several participant‐level summary statistics to estimate overall deviation from the normative pattern.

By applying the free‐water model, we demonstrated that the dMRI signal holds more information regarding schizophrenia pathologies than the FA measure. Both the FAt and FW measures had overall better predictive power than the FA measure alone, suggesting that the increased specificity provided by the more elaborated free‐water model is able to identify features that are more directly contributing to the separation between individuals diagnosed with schizophrenia and healthy controls. Additionally, including both FAt and FW together had the best predictive power. The improvement in predictive power compared to each measure on its own, suggests that accounting for the co‐occurrence of two or more pathologies is also important for the characterization of schizophrenia. This is in line with previous free‐water studies that identified variable rates of FAt and FW abnormalities along the different stages of schizophrenia (Lyall et al., 2018; Oestreich et al., 2017; Pasternak et al., 2015; Pasternak, Westin, et al., 2012; Tang et al., 2019), further supporting the hypothesis that each measure accounts for a different pathology. Finally, the application of the free‐water model resulted in differences between males and females with respect to the best predictors. This is aligned with previous studies which observed sexually‐dimorphic free water increase, which was suggested to be the result of an increased acute response in the female subjects diagnosed with schizophrenia relative to male subjects (Lyall et al., 2018). We note, however, that even though these findings may suggest different abnormality patterns between the sexes, they might as well be the result of differences in the number of subjects of each sex (659 males, 454 females) in our data, or due to the different proportions of subjects belonging to the control group versus subjects belonging to the schizophrenia group (279:380 in males, 233:221 in females), and therefore requires further research.

We note that previous studies showed that the type and extent of FAt and FW abnormalities depend on age, and on the stage of the disorder (e.g., prodromal, first‐psychotic episode, early psychosis, and chronic) (Pasternak, Kelly, Sydnor, & Shenton, 2018). Therefore, the current data, that are heterogeneous in terms of disorder stage, may not be optimal for the identification of predictive clinical features. Nevertheless, the acceptable level of predictive power is expected to increase when the same methods are applied to datasets that are clinically more homogenous.

Our findings show that the combination of multiple imaging features increases the predictive performance of the model. This suggests that it would be beneficial to include additional measures of interest, for example, more elaborate dMRI models, clinical phenotypes, or volumetric/cortical thickness measures, and develop more elaborate normative models that combine information from more than one feature at a time, to further improve prediction performance. , In this study we focused on the prediction of single‐subject classification (i.e., schizophrenia or control) where we used regularized ridge regression. The choice of this binary classifier, together with the relatively large sample size, considerably reduced the risk of overfitting (Arbabshirani et al., 2017). However, the use of more elaborate machine‐learning models (Ardekani et al., 2011; Chand et al., 2020; Lee et al., 2018; Mikolas et al., 2018; Srinivasagopalan, Barry, Gurupur, & Thankachan, 2019) could also be considered in order to increase further the predictive performance. Availability of clinical parameters may also generalize our approaches to the prediction of other properties, such as clinical outcome, or treatment response. We anticipate that using normative models will improve performance of such prediction models as well.

An additional contribution of this article is our novel approach to controlling for confounders, namely, age and sex. Our approach mainly differs from recent studies using normative modeling (Bouix et al., 2013; Chamberland et al., 2020; Dean III et al., 2017; Dimitrova et al., 2020; Lv et al., 2020; Marquand et al., 2016; Pasternak et al., 2014; Taylor et al., 2020; Wolfers et al., 2018) by our consideration of sex in an exact‐matching way, rather than as an additional covariate. Our approach for controlling for age is similar to other studies using nonparametric methods for the modeling of the normative range, see for example, (Marquand et al., 2019) for a review. Most common methods for adjusting for age and sex assume the dependency has a functional form, for example, linear, which may be either an over‐simplification or over‐fitting, depending on the complexity of the functional form. In turn, mis‐modeling the dependency of age and sex could result in bias or noise that could cause false positive and false negative findings. Our method is nonparametric, and, similar to Wolfers et al. (2018), is therefore not only robust but it does not rely on any assumptions on the functional form. The use of a leave‐one‐out approach for choosing the bandwidth also allows for better control of the confounding variables, and makes it possible to identify ROIs that do not necessarily need to be adjusted. While in the ideal situation of infinitely many healthy controls, the best way to control for age and sex would be to model the normative range for every subject by only considering healthy controls that exactly match the subject's covariates—Our method builds on the idea of exact matching but is also suitable for finite sample sizes, where an infinite size of healthy control population is not available. We note that the fact that the individual deviations provided better effect sizes and predictive power than the raw values could also be attributed to the inherently more accurate control for age/sex that was applied in the calculation of the deviations.

This study nonetheless has several limitations. First, since the dMRI data from this study were retrospectively harmonized, they were not acquired with state‐of‐the‐art acquisition protocols. A more current protocol with multiple *b*‐value shells and better image resolution would improve the accuracy of the bi‐tensor model fit (Pasternak, Shenton, & Westin, 2012). Second, the analysis we performed did not account for the data heterogeneity in the context of different treatment protocols and different comorbid substance use/abuse, which may serve as possible confounders of our results. In addition, as previous studies (Hill et al., 2013; Reininghaus et al., 2019; Skudlarski et al., 2013; Tamminga et al., 2013) show that the abnormality pattern observed in schizophrenia overlaps with the abnormality pattern observed in other psychotic disorders, it is a matter of future research to test the specificity of our findings to schizophrenia. Lastly, investigating the relationship between clinical symptoms and the brain abnormalities found is beyond the scope of the current article, but serves as an important avenue for future studies.

In conclusion, our findings suggest several important insights to subject‐level classification methods and their utility in schizophrenia. First, normative modeling approaches may improve subject‐level predictions. Second, setting a “normal” threshold and using only those deviations that exceed this threshold derives summary measures that are limited in their ability to perform predictions. Rather, the interplay between the individual deviations across different fiber tracts is preferred. Third, splitting FA values into FAt and FW contributions may improve the group separation of healthy controls and schizophrenia. Taken together these conclusions imply that schizophrenia is highly likely to be characterized by subtle changes in white matter microstructure that are distributed across brain locations, rather than characterized by severe focal lesions.

## CONFLICT OF INTEREST

The authors declare that they have no conflict of interest.

## ETHICS APPROVAL AND PATIENT CONSENT

All data, except for the Philadelphia Neurodevelopmental Cohort (PNC) (Satterthwaite et al., 2014, 2016) were provided by the principal investigators following procurement of Institutional Review Board approvals for sharing and analyzing de‐identified data. PNC data were downloaded from the National Institute of Health (NIH) database following NIH approval.

## Supporting information

**Supplementary Table S1** Chosen bandwidth for each modality and ROI
**Supplementary Table S2***p*‐values and Cohen's‐*d* effect sizes for a 1‐tailed *t* test searching for lower values in the schizophrenia patients group in FA and FAt modalities, and for higher values in the schizophrenia patients group in the FW modality. Significant values (*p* < .05) are marked in a bold font.
**Supplementary Table S3** Fraction of individuals with abnormal *z*‐scores in each ROI (infra‐normal in FA, FAt and supra‐normal in FW)

**Supplementary Figure S1** Effect sizes (in absolute values) obtained when testing for group differences in each of the summary measures, in FA (green bars), FAt (red bars), and FW (blue bars). The acronyms are identical to the ones used in Figure 2.
*.01 < *p* < .05, **.001 < *p* < .01, ***p < .001

**Supplementary Figure S2** Area under the receiver–operator curves (AUC), averaged over the cross‐validations, in each of the ROIs, for FA (green bars), FAt (red bars), and FW (blue bars)

**Supplementary Figure S3** Box plot of the logistic regression coefficients, across the cross‐validations, obtained when the FAt and FW *z*‐scores in all ROIs are inserted simultaneously as an input

**Supplementary Figure S4** Area under the receiver–operator curves (AUC), averaged over the cross‐validations, obtained when inputting the values in all ROIs simultaneously into the classifier, for each sex separately

## Data Availability

Research data are not shared.
