# Supplementary material for: Improving the predictive potential of diffusion MRI in schizophrenia using normative models—Towards subject‐level classification
Source: Hum Brain Mapp. 2021 Jul 29;42(14):4658–70. doi: 10.1002/hbm.25574 (PMC8410550; doi:10.1002/hbm.25574)

Acronyms used in the text for the fiber tracts

| Fmajor = Forceps major |
| --- |
| Fminor = Forceps minor |
| Fornix |
| Lcing = Left cingulum – hippocampal |
| Lcing2 = Left cingulum – cingulate gyrus |
| Lcst = Left corticospinal tract |
| Lifo = Left inferior fronto-occipital fasciculus |
| Lilf = Left inferior longitudinal fasciculus |
| Lslf = Left superior longitudinal fasciculus |
| Lunc = Left uncinate |
| Rcing = Right cingulum – hippocampal |
| Rcing2 = Right cingulum – cingulate gyrus |
| Rcst = Right corticospinal tract |
| Rifo = Right inferior fronto-occipital fasciculus |
| Rilf = Right inferior longitudinal fasciculus |
| Rslf = Right superior longitudinal fasciculus |
| Runc = Right uncinate |
| WM skeleton = white matter skeleton |

Supplementary table 1 – chosen bandwidth for each modality and ROI

|  | **FA** | **FAt** | **FW** |
| --- | --- | --- | --- |
| Fmajor | 7 | 9 | 5.5 |
| Fminor | 2.5 | 2 | 2.5 |
| Fornix | 5.5 | 5.5 | 3 |
| Lcing | 4 | 10 | 3 |
| Lcing2 | 4.5 | 8.5 | 12.5 |
| Lcst | 2 | 1.5 | 2.5 |
| Lifo | 8 | 9.5 | 2 |
| Lilf | 9.5 | 6.5 | 6.5 |
| Lslf | 12.5 | 72 | 6 |
| Lunc | 10 | 8.5 | 72 |
| Rcing | 4 | 6 | 3 |
| Rcing2 | 5 | 15.5 | 6 |
| Rcst | 1.5 | 1.5 | 2 |
| Rifo | 2.5 | 10.5 | 2 |
| Rilf | 8 | 9.5 | 5.5 |
| Rslf | 6 | 14 | 5 |
| Runc | 10 | 8 | 7.5 |
| WM skeleton | 2 | 2 | 2 |

Supplementary Table 2 – p-values and Cohen's-d effect sizes for a 1-tailed t-test searching for lower values in the schizophrenia patients group in FA and FAt modalities, and for higher values in the schizophrenia patients group in the FW modality. Significant values (p<0.05) are marked in a bold font.

|  | | **FA** | | | **FAt** | | **FW** | |
| --- | --- | --- | --- | --- | --- | --- | --- | --- |
|  |  | raw | | z | raw | z | raw | z |
| Fmajor | p-value | **2.7e-13 0.43** | **5.2e-14 0.44** | | **2.3e-11 0.39** | **5e-10 0.37** | **2.2e-17 -0.5** | **2e-19 -0.53** |
|  | Cohen's d |  |  |  |  |  |  |  |
| Fminor | p-value | **6e-10 0.36** | **2.2e-11 0.4** | | **2.8e-12 0.417** | **1.2e-11 0.4** | **4.3e-6 -0.26** | **4.4e-9 -0.34** |
|  | Cohen's d |  |  |  |  |  |  |  |
| Fornix | p-value | **4e-11 0.39** | **1.4e-12 0.42** | | **7.5e-10 0.36** | **2.16e-10 0.37** | **7.4e-12 -0.4** | **1.5e-11 -0.4** |
|  | Cohen's d |  |  |  |  |  |  |  |
| Lcing | p-value | 0.2 0.05 | **0.015** **0.13** | | **9.5e-4 0.18** | **1.3e-4 0.22** | 0.76 -0.04 | 0.1 -0.07 |
|  | Cohen's d |  |  |  |  |  |  |  |
| Lcing2 | p-value | **0.017 0.12** | **2.9e-4 0.2** | | **3e-6 0.27** | **5e-7 0.29** | 0.24 -0.04 | 0.15 -0.06 |
|  | Cohen's d |  |  |  |  |  |  |  |
| Lcst | p-value | 0.87 -0.06 | 0.68 -0.02 | | 0.77 -0.04 | 0.85 -0.06 | 0.9 0.07 | 0.65 0.02 |
|  | Cohen's d |  |  |  |  |  |  |  |
| Lifo | p-value | **0.001 0.17** | **2.5e-4 0.2** | | **3e-6 0.27** | **5.4e-6 0.26** | 0.17 -0.05 | **0.01 -0.13** |
|  | Cohen's d |  |  |  |  |  |  |  |
| Lilf | p-value | **0.0002 0.21** | **4.6e-4 0.2** | | **5.4e-5 0.23** | **0.001 0.18** | **0.004 -0.15** | **7.4e-4 -0.19** |
|  | Cohen's d |  |  |  |  |  |  |  |
| Lslf | p-value | **0.005 0.15** | **0.0051 0.15** | | 0.08 0.08 | 0.1 0.07 | **3e-8 -0.32** | **1.9e-10 -0.37** |
|  | Cohen's d |  |  |  |  |  |  |  |
| Lunc | p-value | **0.008 0.14** | **0.0024 0.17** | | **5.3e-4 0.19** | **9.8e-4 0.18** | 0.08 -0.08 | **0.04 -0.1** |
|  | Cohen's d |  |  |  |  |  |  |  |
| Rcing | p-value | **0.02 0.12** | **7.6e-5 0.23** | | **1.6e-4 0.21** | **4.2e-6 0.27** | 0.57 0.01 | 0.132 -0.06 |
|  | Cohen's d |  |  |  |  |  |  |  |
| Rcing2 | p-value | **0.005 0.15** | **8.7e-5 0.22** | | **8e-7 0.28** | **1.1e-7 0.31** | **0.02 -0.12** | **0.0012 -0.18** |
|  | Cohen's d |  |  |  |  |  |  |  |
| Rcst | p-value | 0.92 -0.08 | 0.63 -0.02 | | 0.84 -0.06 | 0.81 -0.05 | 0.8 0.06 | 0.38 -0.01 |
|  | Cohen's d |  |  |  |  |  |  |  |
| Rifo | p-value | **0.004 0.15** | **1.9e-4 0.21** | | **3.4e-5 0.23** | **6.6e-5 0.23** | 0.12 -0.07 | **0.002 -0.16** |
|  | Cohen's d |  |  |  |  |  |  |  |
| Rilf | p-value | 0.2 0.04 | 0.1798 0.05 | | 0.09 0.07 | 0.27 0.03 | **0.005 -0.15** | **3.1e-4 -0.2** |
|  | Cohen's d |  |  |  |  |  |  |  |
| Rslf | p-value | 0.1 0.07 | **0.0096 0.14** | | 0.11 0.07 | 0.06 0.09 | **0.001 -0.18** | **1.19e-6 -0.28** |
|  | Cohen's d |  |  |  |  |  |  |  |
| Runc | p-value | 0.26 0.03 | 0.15 0.06 | | 0.05 0.09 | 0.104 0.07 | 0.26 -0.03 | 0.1 -0.07 |
|  | Cohen's d |  |  |  |  |  |  |  |
| WM skeleton | p-value | **2.7e-9 0.35** | **9.8e-11 0.39** | | **1e-11 0.4** | **1.5e-11 0.41** | **2.4e-8 -0.33** | **5e-14 -0.45** |
|  | Cohen's d |  |  |  |  |  |  |  |

Supplementary Table 2* – p-values and Cliff's delta effect sizes for a 1-tailed Wilcoxon rank-sum test searching for lower values in the schizophrenia patients group in FA and FAt z-scores, and for higher values in the schizophrenia patients group in the FW z-scores. Significant values (p<0.05) are marked in a bold font.

|  | | **FA z** | **FAt z** | | **FW z** |
| --- | --- | --- | --- | --- | --- |
| Fmajor | p-value | **1.4e-13 0.25** | | **8.8e-11 0.22** | **3.5e-18 -0.3** |
|  | Cliff's-delta |  |  |  |  |
| Fminor | p-value | **7.3e-13**  **0.24** | | **2.8e-11 0.23** | **1.6e-9 -0.2** |
|  | Cliff's-delta |  |  |  |  |
| Fornix | p-value | **1.3e-13**  **0.25** | | **1.1e-8 0.19** | **1.4e-11 -0.23** |
|  | Cliff's-delta |  |  |  |  |
| Lcing | p-value | **0.008**  **0.08** | | **1.7e-4 0.12** | 0.21 -0.02 |
|  | Cliff's-delta |  |  |  |  |
| Lcing2 | p-value | **1.3e-4**  **0.12** | | **5.8e-7 0.16** | 0.2 -0.02 |
|  | Cliff's-delta |  |  |  |  |
| Lcst | p-value | 0.53 -0.003 | | 0.93 -0.05 | 0.27 -0.02 |
|  | Cliff's-delta |  |  |  |  |
| Lifo | p-value | **1.1e-4 0.12** | | **1.9e-5 0.14** | **0.01 -0.07** |
|  | Cliff's-delta |  |  |  |  |
| Lilf | p-value | **3.8e-4 0.11** | | **0.001 0.1** | **3.4e-4 -0.11** |
|  | Cliff's-delta |  |  |  |  |
| Lslf | p-value | **0.01 0.08** | | 0.16 0.03 | **1e-9 -0.21** |
|  | Cliff's-delta |  |  |  |  |
| Lunc | p-value | **5.3e-4 0.11** | | **0.001 0.1** | 0.1 -0.04 |
|  | Cliff's-delta |  |  |  |  |
| Rcing | p-value | **3e-5 0.13** | | **1.7e-6 0.16** | 0.09 -0.04 |
|  | Cliff's-delta |  |  |  |  |
| Rcing2 | p-value | **5.4e-5 0.13** | | **2.4e-7 0.17** | **2.3e-4 -0.12** |
|  | Cliff's-delta |  |  |  |  |
| Rcst | p-value | 0.69 -0.01 | | 0.93 -0.05 | 0.23 -0.02 |
|  | Cliff's-delta |  |  |  |  |
| Rifo | p-value | **8.8e-6 0.15** | | **8.3e-5 0.13** | **1.1e-4 -0.13** |
|  | Cliff's-delta |  |  |  |  |
| Rilf | p-value | 0.2 0.02 | | 0.35 0.01 | **5.5e-5 -0.13** |
|  | Cliff's-delta |  |  |  |  |
| Rslf | p-value | **0.004 0.09** | | 0.13 0.03 | **1.8e-6 -0.16** |
|  | Cliff's-delta |  |  |  |  |
| Runc | p-value | 0.08 0.04 | | 0.07 0.05 | 0.08 -0.04 |
|  | Cliff's-delta |  |  |  |  |
| WM skeleton | p-value | **3e-16 0.28** | | **1e-14 0.27** | **1.4e-15 -0.27** |
|  | Cliff's-delta |  |  |  |  |

Supplementary Table 3 – fraction of individuals with abnormal z-scores in each ROI (infra-normal in FA,FAt and supra-normal in FW)

|  | **FA controls patients** | | **FAt controls patients** | | **FW controls patients** | |
| --- | --- | --- | --- | --- | --- | --- |
| Fmajor | 1.17% | 3.16% | 0.97% | 1.99% | 0.78% | 3.99% |
| Fminor | 1.56% | 1.49% | 0.19% | 1.66% | 1.36% | 3.16% |
| Fornix | 0.78% | 1.16% | 1.56% | 5.49% | 0 | 0 |
| Lcing | 0.97% | 1.16% | 0.78% | 0.83% | 0.58% | 1.49% |
| Lcing2 | 0.78% | 1.33% | 0.58% | 1.33% | 0.58% | 0.49% |
| Lcst | 0.58% | 0.83% | 1.56% | 1.16% | 0. 97% | 0 |
| Lifo | 1.36% | 0.99% | 0.58% | 1.66% | 1.17% | 1.49% |
| Lilf | 0.19% | 0% | 0.19% | 0.66% | 0.78% | 0.66% |
| Lslf | 0 | 0.83% | 0.19% | 1.16% | 0.78% | 3.32% |
| Lunc | 0.78% | 0.49% | 0.39% | 0.998% | 0.78% | 0.99% |
| Rcing | 0.78% | 0.83% | 0.58% | 1.99% | 1.56% | 0.99% |
| Rcing2 | 0.97% | 1.66% | 0.19% | 1.99% | 0.78% | 1.99% |
| Rcst | 1.56% | 0.49% | 0.97% | 0.83% | 1.17% | 0.33% |
| Rifo | 0.78% | 0.33% | 0.78% | 0.66% | 0.97% | 1.16% |
| Rilf | 0.39% | 0% | 0.19% | 0.49% | 1.17% | 1.16% |
| Rslf | 0.39% | 0.49% | 0.19% | 0.99% | 0.97% | 2.82% |
| Runc | 0.58% | 0.16% | 0.19% | 0.33% | 0.78% | 0.83% |
| WM skeleton | 1.95% | 1.49% | 1.17% | 2.16% | 0.78% | 2.82% |

Supplementary Figure 1- Effect sizes (in absolute values) obtained when testing for group differences in each of the summary measures, in FA (green bars), FAt (red bars), and FW (blue bars).The acronyms are identical to the ones used in Figure 2.
* 0.01<p<0.05, ** 0.001<p<0.01, *** p<0.001
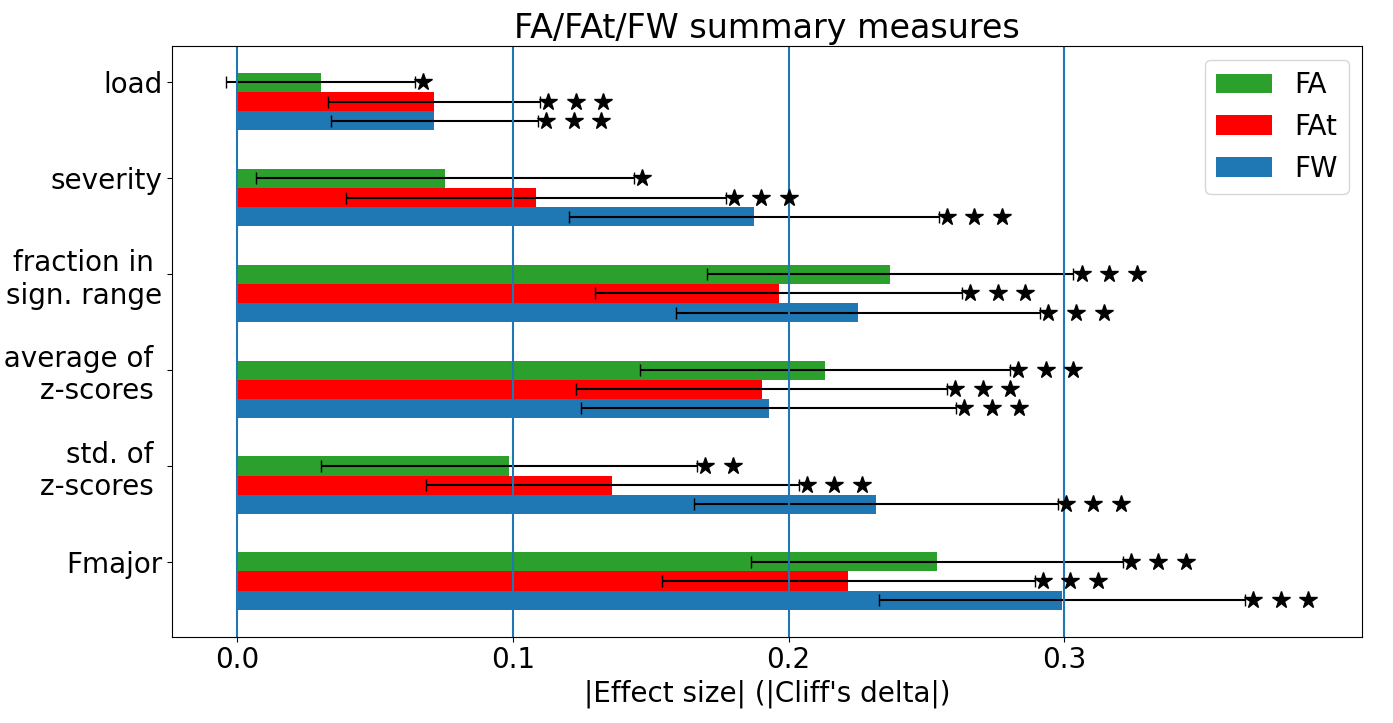


Supplementary Figure 2- Area under the receiver-operater curves (AUC), averaged over the cross-validations, in each of the ROIs, for for FA (green bars), FAt (red bars), and FW (blue bars).


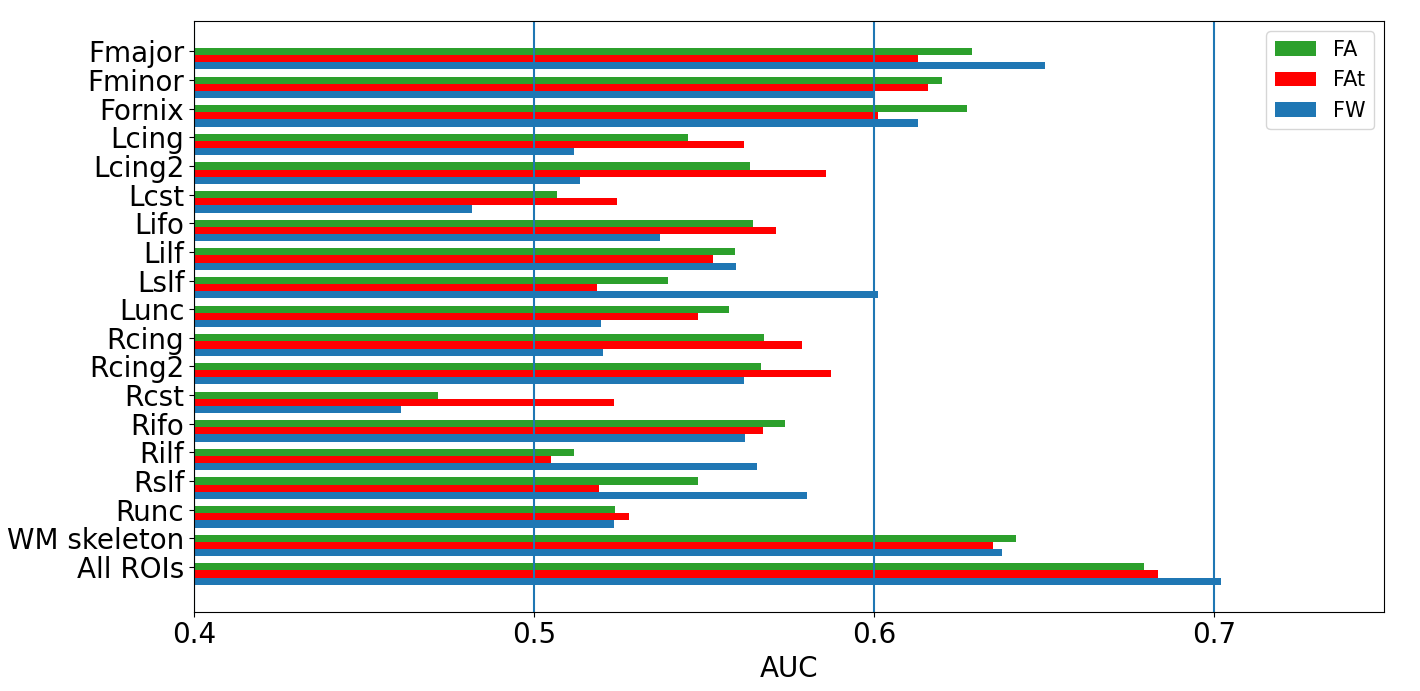


Supplementary Figure 3 – Box plot of the logistic regression coefficients, across the cross-validations, obtained when the FAt and FW z-scores in all ROIs are inserted simultaneously as an input.


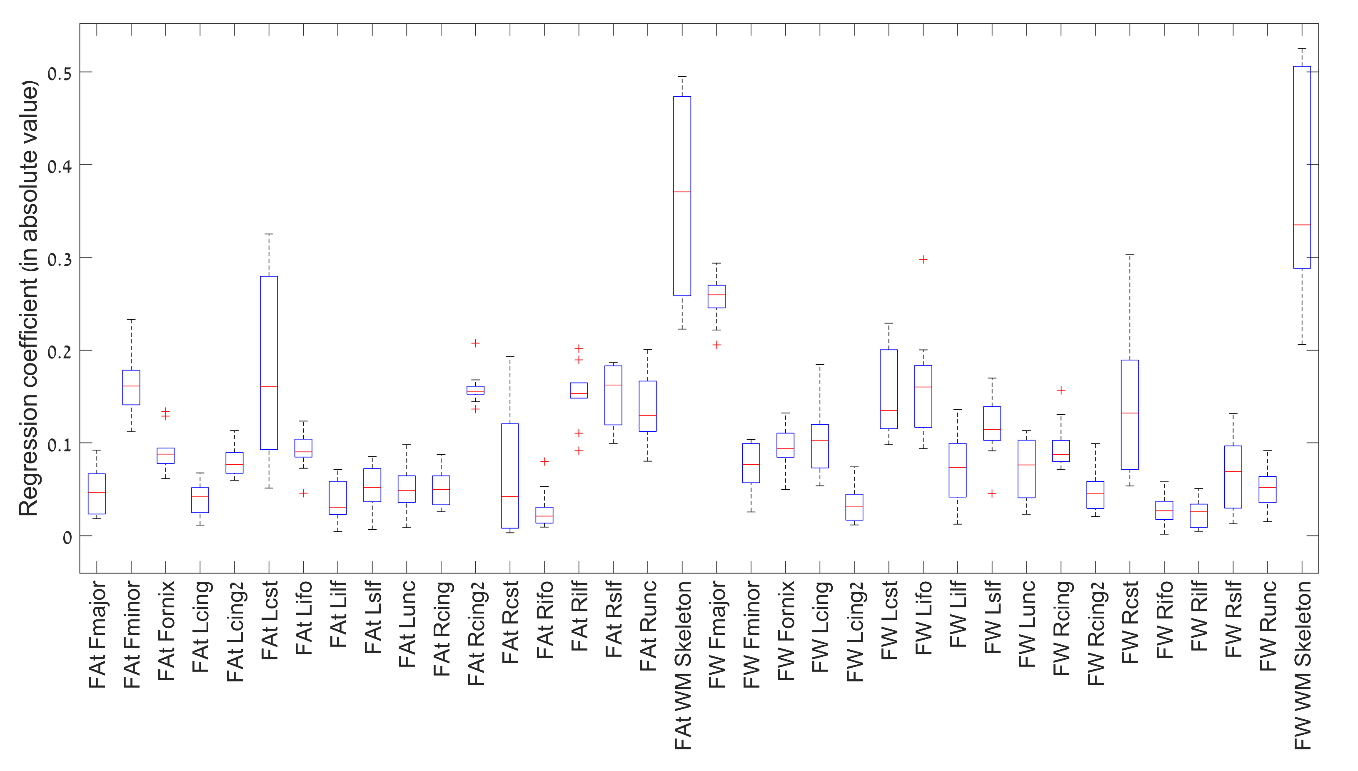


Supplementary Figure 4- Area under the receiver-operater curves (AUC), averaged over the cross-validations, obtained when inputting the values in all ROIs simuoultaneously into the classifier, for each sex separately.


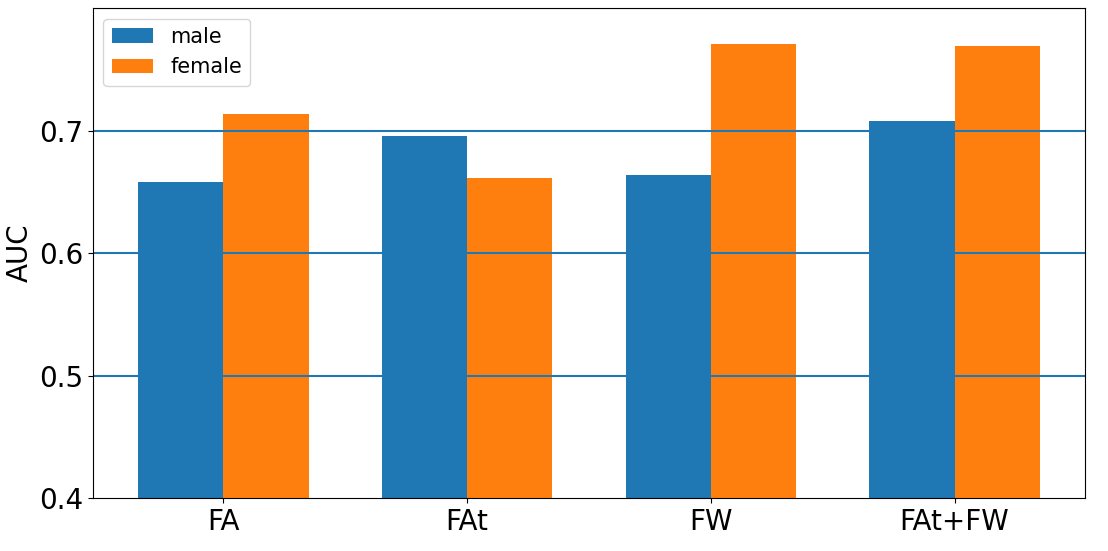

Supplement: Supplementary file 1 — Supplementary Table S1 Chosen bandwidth for each modality and ROI Supplementary Table S2p‐values and Cohen's‐d effect sizes for a 1‐tailed t test searching for lower values in the schizophrenia patients group in FA and FAt modalities, and for higher values in the schizophrenia patients group in the FW modality. Significant values (p < .05) are marked in a bold font. Supplementary Table S3 Fraction of individuals with abnormal z‐scores in each ROI (infra‐normal in FA, FAt and supra‐normal in FW) [file HBM-42-4658-s002.docx]
