# Supplementary figures and images for: Improving the predictive potential of diffusion MRI in schizophrenia using normative models—Towards subject‐level classification
Source: Hum Brain Mapp. 2021 Jul 29;42(14):4658–70. doi: 10.1002/hbm.25574 (PMC8410550; doi:10.1002/hbm.25574)

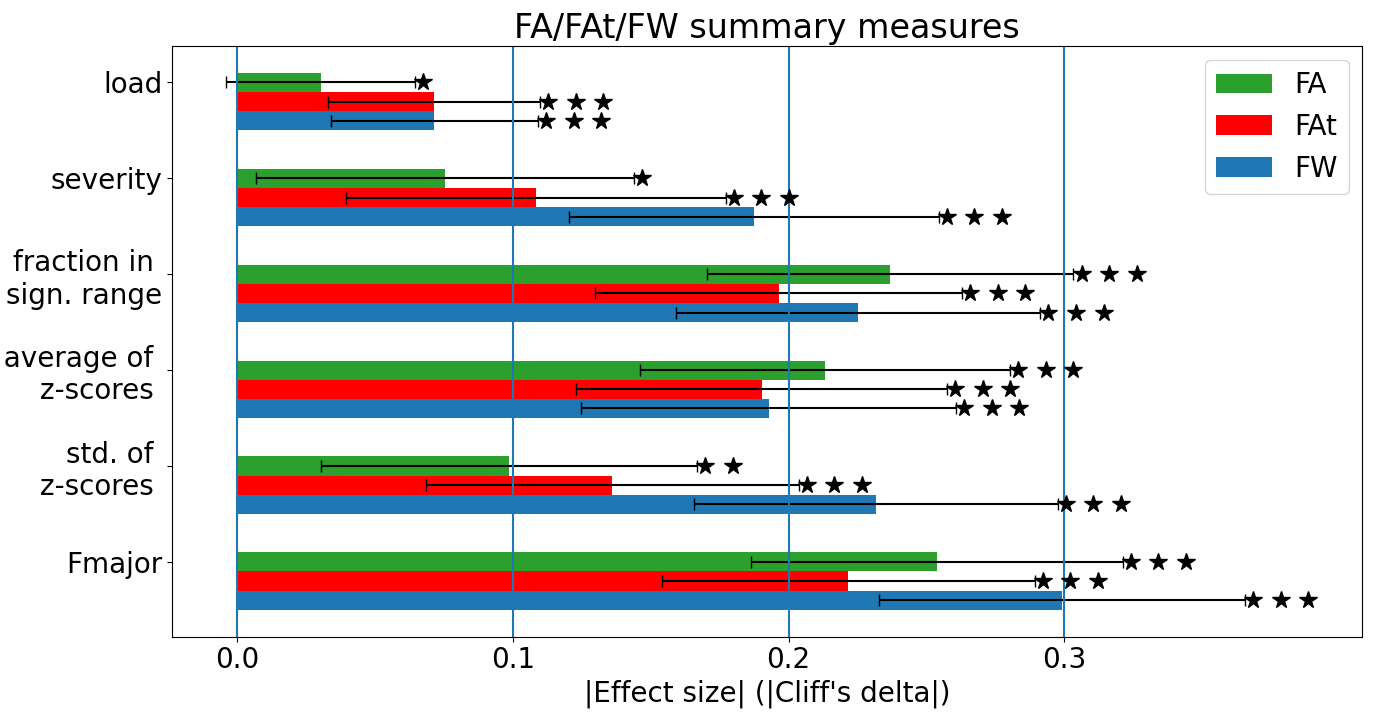

Supplement: Supplementary file 2 — Supplementary Figure S1 Effect sizes (in absolute values) obtained when testing for group differences in each of the summary measures, in FA (green bars), FAt (red bars), and FW (blue bars). The acronyms are identical to the ones used in Figure 2. *.01 < p < .05, **.001 < p < .01, ***p < .001 [file HBM-42-4658-s001.png]

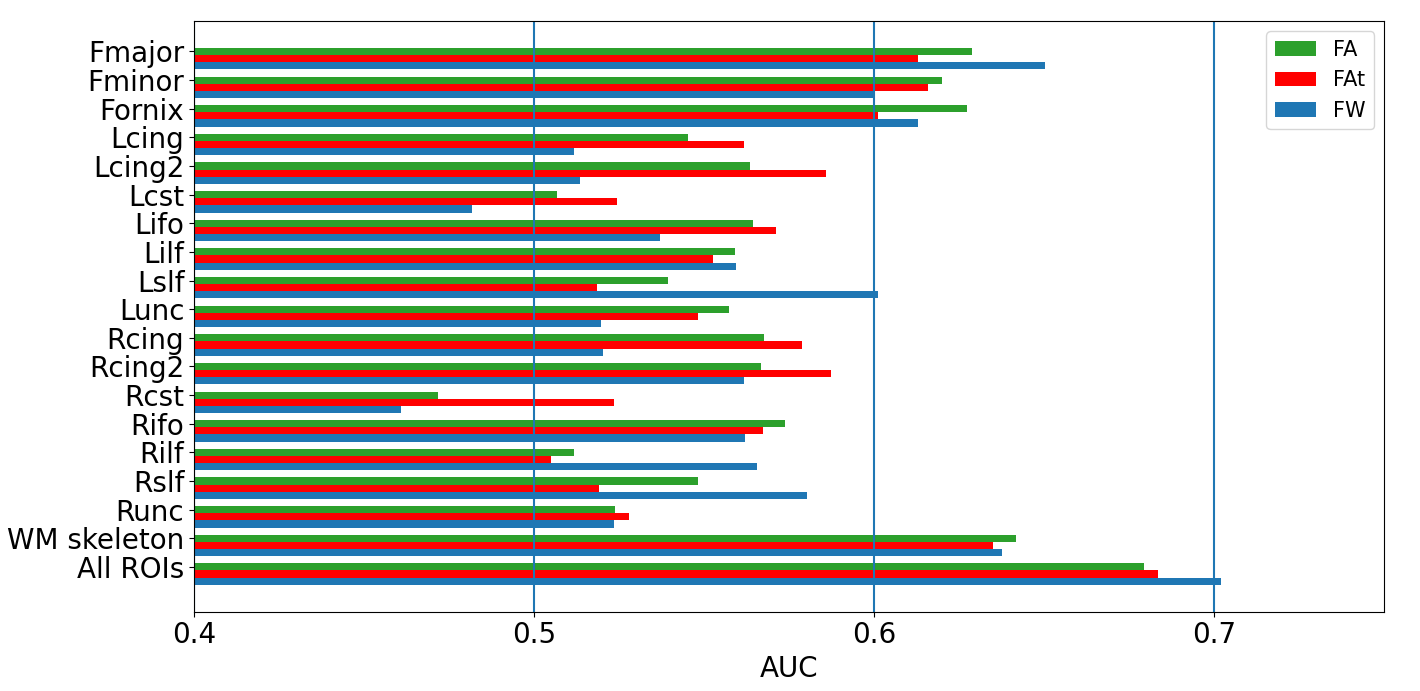

Supplement: Supplementary file 3 — Supplementary Figure S2 Area under the receiver–operator curves (AUC), averaged over the cross‐validations, in each of the ROIs, for FA (green bars), FAt (red bars), and FW (blue bars) [file HBM-42-4658-s004.png]

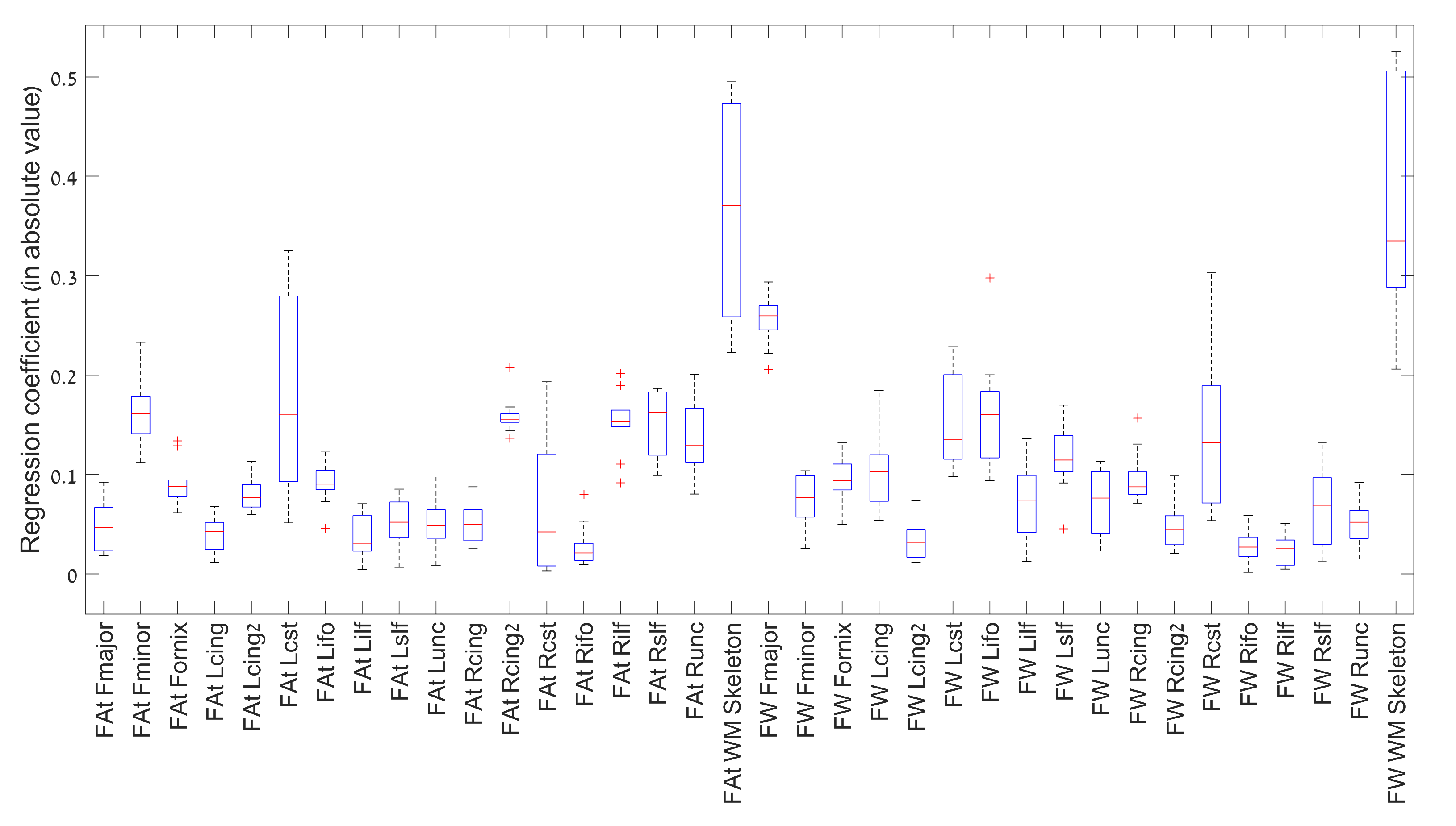

Supplement: Supplementary file 4 — Supplementary Figure S3 Box plot of the logistic regression coefficients, across the cross‐validations, obtained when the FAt and FW z‐scores in all ROIs are inserted simultaneously as an input [file HBM-42-4658-s005.tif]

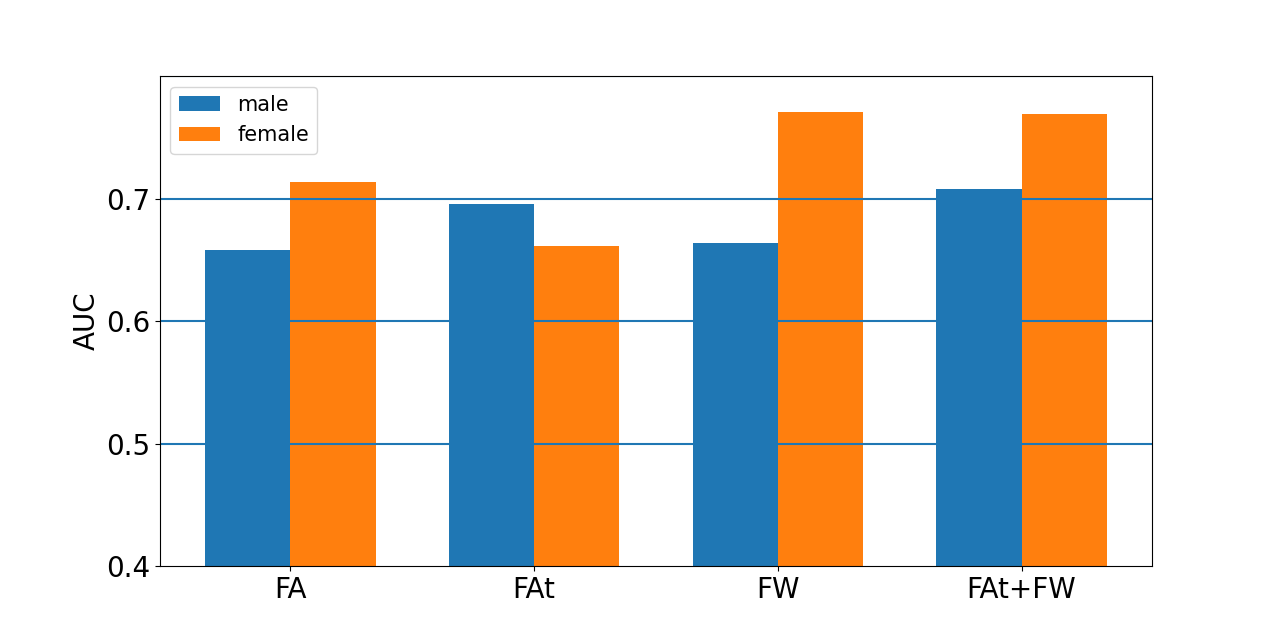

Supplement: Supplementary file 5 — Supplementary Figure S4 Area under the receiver–operator curves (AUC), averaged over the cross‐validations, obtained when inputting the values in all ROIs simultaneously into the classifier, for each sex separately [file HBM-42-4658-s003.png]
